# The safty profile of blood salvage applied for collected blood with amniotic fluid during cesarean section

**DOI:** 10.1186/s12884-022-04488-3

**Published:** 2022-02-28

**Authors:** Xiaoying Rong, Xiangyang Guo, Hong Zeng, Jun Wang, Mi Li, Yang Wang

**Affiliations:** Department of Anesthesiology, Peking University Third Hospital, Beijing, China

**Keywords:** Cell salvage, Amniotic fluid, Obstetrics

## Abstract

**Background:**

The guidelines of National Health Service(NHS, the United Kingdom) recommended for use in obstetrics at increased risk of bleeding, requiring two suction devices to reduce amniotic fluid contamination, however, when comes to massive hemorrhage, it is may difficult to operate because the complex operation may delay time. The aim of the study was to detect the effect of amniotic fluid recovery on intraoperative cell salvage in obstetrics and provide evidence for clinical applications.

**Method:**

Thirty-four patients undergoing elective cesarean section were randomly divided into two groups. In group 1, the cumulative blood from the operation field, including the amniotic fluid, was collected using a single suction device for processing. In group 2, after suctioning away the amniotic fluid using another suction device for the cumulative blood from the operation field. From each group, four samples were taken, including maternal venous blood (sample I), blood before washing (sample II), blood after washing (sample III) and blood after filtration with a leukocyte filter (sample IV), to detect serum potassium (K +), hemoglobin (Hb), white blood cell (WBC), fetal hemoglobin (HbF), alpha fetoprotein (AFP) and squamous cell (SC) levels.

**Results:**

The AFP, K + and WBC levels of sample III and sample IV were significantly lower than sample I in group 1 and group 2 (*P* < 0.05). Significantly more SCs were found in sample III than in sample I in group 1 and group 2 (*P* < 0.05), but SCs of sample IV had no statistical difference compared to sample I in group 1 and group 2 (*P* > 0.05). There was no significant difference in the K + , Hb, WBC, AFP and SC levels of sample IV between group 1 and group 2 (*P* > 0.05). The HbF levels of sample III and sample IV were significantly higher in group 1 than in group 2 (*P* < 0.05).

**Conclusion:**

There is little or no possibility for AF contamination to enter the re-infusion system when used in conjunction with a leucodepletion filter. For maternal with Rh-negative blood, we recommend two suction devices to reduce HbF pollution.

**Trial registration:**

ChiCTR1800015684, 2018.4.15.

## Background

Due to concerns that Intraoperative cell salvage(IOCS) in cesarean section may cause amniotic fluid-contaminated blood transfusions, which may lead to amniotic fluid embolism (AFE), the routine use of IOCS in caesarean section is debatable. The Association of Anaesthetists 2018 guideline on cell salvage for peri-operative blood conservation states’the Working Party recommends that cell salvage is not used routinely based on the current evidence, for elective, urgent or emergency caesarean section’[1]. The guidelines of National Health Service(NHS) also recommended for use in obstetrics at increased risk of bleeding, requiring two suction devices to reduce amniotic fluid contamination, however, when comes to massive hemorrhage, it is may difficult to operate because the complex operation may delay time. The aim of the study was to detect the effect of amniotic fluid recovery on intraoperative cell salvage in obstetrics and provide evidence for clinical applications.

## Methods

The clinical study protocol was approved by the Ethics Committee of Peking University Third Hospital. All patients signed an informed consent form before participating in this study The sample size was calculated according to a preliminary experiment of 20 patients with squamous cell (SC) and alpha fetoprotein (AFP) levels as the main indexes. A paired sample t-test (nonparametric calibration) was performed with alpha = 0.05 and beta = 0.2. Thirty-four cases undergoing elective cesarean section from October 2014 to January 2015 in Peking University Third Hospital were randomly divided into two groups. In group 1, the cumulative blood from the operation field, including the amniotic fluid, was collected using a suction device for processing with the Haemonetics-5 Cell Saver system. In group 2, using two suction devices, after suctioning away the amniotic fluid, the cumulative blood from the operation field was collected using another suction device for processing with Haemonetics-5 Cell Saver system. From each group, four samples were taken, including maternal venous blood (sample I), blood before washing (sample II), blood after washing (sample III) and blood after filtration with a leukocyte filter (sample IV), to detect serum potassium (K +), hemoglobin (Hb), white blood cell (WBC), fetal hemoglobin (HbF), AFP and SC levels.

After the pre-test of 20 patients, using squamous cells and alpha-fetoprotein as the main indicators, the sample size was calculated by paired-sample t-test (non-parametric correction), set alpha = 0.05, beta = 0.2, and calculated by PASS. The sample size is 34. Statistical analysis was performed with SPSS 19.0. All continuous nonparametric data are described as the mean ± SD or the median (interquartile range). In-group comparisons were performed using the relevant sample Friedman analysis, and the comparison between groups was performed using the independent sample Mann–Whitney test.*P* < 0.05 was considered statistically significant.

## Results

Patients in group 1 and 2 had an average age of 31 ± 4 years and 32 ± 2 years, respectively; the gestational age in group 1 and group 2 was 37 ± 2 weeks and 38 ± 2 weeks. The two groups showed no difference in age or gestational age.Table 1 gives the reason for Caesarean section.

**Table 1:** Reasons for Caesarean section

Reason	Number
Breech	7
Previous section	6
twins	6
Placenta	6
fetal macrosomia	4
contracted pelvic	2
protracted active phase	2
Placenta praevia	1

The average K + , AFP, HbF, WBC, Hb and SC levels of sample I, sample II, sample III and sample IV for group 1 and group 2 are shown in Tables 2 and 3, along with statistical analysis.

**Table 2:** Average K + , AFP, HbF, WBC, Hb and SC levels in group 1

	sample I	sample II	sample III	sample IV
K + (mmol/L)	3.57 (3.38–3.91)	3.33 (2.73–4.22)	1.98 (1.29–2.23)	1.82 (1.065–2.09)^a^
AFP (μg/L)	239.4 (151.05–361.3)	473 (145.9–1210)	3.5 (145.9–1210)^a^	3.82 (145.9–1210)^a^
HbF (%)	0.7 (0.5–0.95)	1.2 (0.3–8.4)	1.9 (0.9–4.15)^c^	1.9 (0.9–4)
WBC (^a^10^9/L)	7.9 (7.19–8.065)	1.13 (0.815–1.425)^c^	2.47 (0.815–1.425)^d^	0.12 (0.09–0.73)^c^
Hb (g/L)	112 (98–120)	35 (18.5–45.5)^b^	114 (76.5–185.5)	93 (65.5–165)^e^
SC (count/μl)	0 (0–0)	2 (1–8.5)^c^	7 (1.5–45)^c^	0 (0–1.5)^d^

Values expressed as medians (twenty-fifth to seventy-fifth percentile)

^a^*P* < 0.05 compared with sample II and sample I

^b^*P* < 0.05 compared with sample I and sample III

^c^*P* < 0.05 compared with sample I

^d^*P* < 0.05 compared with sample III

^e^*P* < 0.05 compared with sample II

**Table 3:** The average K + , AFP, HbF, WBC, Hb and SC levels in group 2

	sample I	sample II	sample III	sample IV
K + (mmol/L)	3.76 (3.36–3.92)	2.76 (2.17–3.33)	1.97 (1.46–2.84)^c^	2.08 (1.435–2.485)^a^
AFP (μg/L)	179.2 (106.08–308.75)	111.9 (83.75–202.95)	1.83(1.055–6.415)^a^	1.51 (1.015–3.985)^a^
HbF (%)	0.5 (0.3–0.8)	0.6 (0–1.2)	1 (0.65–1.3)^c^	0.9 (0.55–1.3)
WBC (^a^10^9/L)	7.47 (4.3–9.53)	1.18 (0.81–1.455)^c^	3.64 (0.81–1.455)	0.36 (0.81–1.455)^b^
Hb (g/L)	107 (92.5–118.5)	33 (19.5–42)^c^	147 (125–190)^a^	135 (110.5–170)^d^
SC (count/μl)	0 (0–0)	0 (0–1)	2 (0–5)^c^	0 (0–0.5)

Values expressed as medians (twenty-fifth to seventy-fifth percentile)

^a^*P* < 0.05 compared with sample II and sample I

^b^*P* < 0.05 compared with sample I and sample III

^c^*P* < 0.05 compared with sample I

^d^*P* < 0.05 compared with sample II

K + , Hb and WBC levels of sample I, sample II, sample III and sample IV had no significant differences between the two groups (*P* > 0.05; Table 4). AFP level of sample II showed statistically higher in Group 1 than in Group 2 (*P* = 0.001), whereas AFP levels of sample I, sample III and sample IV showed no significant differences (*P* > 0.05) between the two groups (Table 5). SC levels of sample II and sample III in Group 1 showed statistically higher than Group 2 (*P* = 0.001 and *P* = 0.012), whereas SC levels of sample I and sample IV showed no significant differences (*P* > 0.05) between the two groups. HbF levels of sample III and sample IV also were higher in Group 1 than in Group 2 (*P* = 0.016 and *P* = 0.012).

**Table 4:** K + , Hb and WBC in Group 1 and Group 2

	Group 1	Group 2	P
K + sample I (mmol/L)	3.57 (3.38–3.91)	3.76 (3.36–3.92)	0.683
K + sample II (mmol/L)	3.33 (2.73–4.22)	2.76(2.17–3.33)	0.057
K + sample III (mmol/L)	1.98 (1.29–2.23)	1.97 (1.46–2.84)	0.375
K + sample IV (mmol/L)	1.82 (1.065–2.09)	2.08 (1.435–2.485)	0.114
Hb sample I (g/L)	112 (98–120)	107 (92.5–118.5)	0.357
Hb sample II (g/L)	35 (18.5–45.5)	33 (19.5–42)	0.734
Hb sample III (g/L)	114 (76.5–185.5)	147 (125–190)	0.394
Hb sample IV (g/L)	93 (65.5–165)	135 (110.5–170)	0.205
WBC sample I (*10^9/L)	7.9 (7.19–8.065)	7.47 (4.3–9.53)	0.474
WBC sample II (*10^9/L)	1.13 (0.815–1.425)	1.18 (0.81–1.455)	0.76
WBC sample III (*10^9/L)	2.47 (0.815–1.425)	3.64 (0.81–1.455)	0.062
WBC sample IV (*10^9/L)	0.12 (0.09–0.73)	0.36 (0.81–1.455)	0.16

Values are expressed as medians (twenty-fifth to seventy-fifth percentile)

*P* < 0.05 was considered statistically significant

**Table 5:** AFP, HbF and SC levels in group 1 and group 2

	Group 1	Group 2	P
AFP sample I (μg/L)	239.4 (151.05–361.3)	179.2 (106.08–308.75)	0.29
AFP sample II (μg/L)	473 (145.9–1210)	111.9 (83.75–202.95)	0.001
AFP sample III (μg/L)	3.5 (145.9–1210)	1.83 (1.055–6.415)	0.357
AFP sample IV (μg/L)	3.82 (145.9–1210)	1.51 (1.015–3.985)	0.231
SC sample I (count/μl)	0 (0–0)	0 (0–0)	0.563
SC sample II (count/μl)	2 (1–8.5)	0 (0–1)	0.001
SC sample III (count/μl)	7 (1.5–45)	2 (0–5)	0.012
SC sample IV (count/μl)	0 (0–1.5)	0 (0–0.5)	0.322
HbF sample I (%)	0.7 (0.5–0.95)	0.5 (0.3–0.8)	0.106
HbF sample II (%)	1.2 (0.3–8.4)	0.6 (0–1.2)	0.131
HbF sample III (%)	1.9 (0.9–4.15)	1 (0–1.2)	0.016
HbF sample IV (%)	1.9 (0.9–4)	0.9 (0–1.2)	0.012

Values are expressed as medians (twenty-fifth to seventy-fifth percentile)

*P* < 0.05 was considered statistically significant

## Discussion

The main components of amniotic fluid include water (98% to 99%); organic components of glucose, fat, protein and protein derivatives, bilirubin, metabolites, fetal AFP, hormones secreted by the placenta and fetus, enzymes (aspartate aminotransferase, alanine aminotransferase, alkaline phosphatase, etc.); inorganic components such as electrolytes; and cells detached from the surface of the fetus. Serum potassium, AFP, tissue factors, and lamellar bodies are considered markers of amniotic fluid [2]. We selected serum potassium, AFP and SCs as markers of amniotic fluid in this study.

This study has shown the efficiency of the washing stage of the cell salvage machine, AF contaminants can be effectively reduced when used in combination with a leucodepletion filter, but amniotic fluid on blood recovery still have some effects on HbF.

Durand et al. found that the cell saver could not completely remove fetal cell debris [3]. Another study showed that in filtered blood samples, AFP was completely removed, while the SC component still existed [4]. Such reports have raised concerns about amniotic fluid embolus (AFE) after autologous blood transfusion. All of the abovementioned studies are consistent with the results of our study, which suggest that SCs require further clearance. In recent years, the concentrations of SCs and lamellar bodies have decreased significantly with the application of leukocyte filters. Elagamy et al. reported that after using a double suction system and a leukocyte filter, median squamous cell counts (0 [0–1] versus 8 [3–12]/high power field) were significantly lower postfiltration compared to prewash values [5]. Sullivan et al. reported similar results [6].

### The leukocyte depletion filter (LDF) effect

Different LDF materials result in different filtration effects on the components of amniotic fluid. Catling et al.[7] reported combining the Haemonetics-5 Cell Saver system (Cell Saver 5 + , Haemonetics Corp., Braintree, MA USA) with a Pall RC 100 LDF (Pall RC 100, Pall Biomedical, Portsmouth, UK). The AFP level was significantly decreased postfiltration, while SCs were not effectively filtered and still existed in nearly half of the filtered samples. Using the same cell saver and the new Pall RS filter (LeukoGuard RS, Pall Biomedical Products Co., East Hills, NY), Waters [8] reported significantly fewer SCs and lamellar bodies.

In this study, a Pall SB leukocyte filter (American blood, LipiGuard SB), which is composed of a polyester non-woven cloth, was used as a filter material. WBCs in sample IV were significantly reduced and almost negligible compared with in sample I and sample III in the two groups. In addition, K + and AFP levels in sample IV were significantly reduced compared with in sample I. SCs in sample IV were significantly reduced compared with sample III, and there was no significant difference compared with sample I, proving that SCs could be effectively removed by LDF, especially collected with amniotic fluid.

### Amniotic fluid on blood recovery effects

Within the last 10 years, the application of intraoperative cell salvage (IOCS) in obstetrics has attracted increasing attention [9–12]. IOCS is already one technique that can be applied to obstetric hemorrhage in the UK; the United States Association of Obstetricians and Gynecologists (ACOG) also promotes the use of IOCS with bleeding due to placenta accrete [13, 14]. In recent discussions on "perioperative blood conservation," the Italy Immunology and Transfusion Medical Commission (SIMIT) recommended that IOCS be used in obstetric emergencies with bleeding or a risk of bleeding, but added leukocyte depletion filtration after the recovery of blood cells [15]. However, these guidelines have not mentioned the effect of amniotic fluid in the recovery of blood loss.

In 1999, Catling et al. [7] divided 27 females into two groups, 13 in whom a single suction device was used for all the blood loss and amniotic fluid. Two suction devices were used in another group of 14 patients, and the suction device was switched to an autologous blood device for blood loss after the amniotic fluid had been completely absorbed by normal suction. After the test, they showed that the use of two suction devices significantly reduced amniotic fluid composition, and in the single-suction device group, SCs were still visible in the recovered blood. In 2008, Sullivan et al. [6] also randomly divided 34 females into two groups. Similar to the results obtained with and without the amniotic fluid, AFP, Hb and SC levels showed no significant differences between prewash, postwash and postfiltration samples. Tanqueray [16] advocated using another suction devices from the time of amniotic membrane rupture until the complete delivery of the fetus and placenta in cesarean sections, in order to minimize amniotic fluid contamination of the collected blood. Nevertheless, at several large maternity units, all intraoperatively lost blood is collected, which improves the volume of RBCs salvaged [17].

In this study, we randomly divided the samples into two groups: A single suction device was used in group 1 for all blood loss in addition to amniotic fluid. Two suction devices were used in group 2 after the beginning of surgery, with an ordinary suction tube used for amniotic fluid until the baby was delivered, and the suction device subsequently switched to an autologous blood recovery device for intraoperative blood loss. We found that AFP in prewash samples, SCs in prewash and postwash samples, and HbF in postwash and postfiltration samples showed significant differences between the two groups. However, there was no significant difference in other samples between the two groups, which confirmed that in addition to HbF, amniotic fluid or no recovery had no significant effect on the components of postfiltration blood.

### HbF and alloimmunization

The cell saver system could not distinguish between fetal and maternal red cells, and thus, any aspirated fetal red cells will be retransfused [9]. Previously, Durand [3] found that the HbF approached 1% in 7 recovered blood samples out of 15 patients, and Rainaldi [18] found that the HbF approached 1.8%, or 2%, in 3 recovered blood samples out of 15 patients. Many studies have confirmed that fetal red blood cells are present in postfiltration blood [7, 8, 19], thereby increasing the risk of maternal alloimmunization when there is incompatibility between maternal and fetal red cell antigens [20].

The neonatal ABO antigen is not well-developed. Because there are few antigen sites, the newborn will produce antibodies after 3 to 6 months after birth. Therefore, the clinical significance of ABO blood group incompatibility is small. Therefore, in the case of maternal and infant blood group incompatibility, it is worth noting that Rh blood type is incompatible. In pregnant females with Rh-negative blood group incompatibility, the maternal anti-D immunoglobulin can neutralize the immune reactions. The dose of anti-D immunoglobulin is mainly based on the matrix determined by the amount of fetal red blood cells.

Catling found that the maximum number of fetal red blood cells in the maternal sample was 19 ml (between 2–19 ml) using IOCS, with 500–2500 IU anti-D immune globulin [8]. In 2011, Ralph used IOCS with LDF in 70 parturients, to evaluate the HbF entered the maternal during transfusing the autologous blood, with a median of 0.8 ml (between 0.2–12.9 ml) [21]. As pregnancy progresses, fetal red blood cells in maternal circulation will increase to more than 2.5 ml in nearly 1% maternal, more than 15 ml in nearly 0.3% materal [22]. Ralph found that the median fetal red blood cell in the maternal circulation before delivery is about 0.48 ml (between 0 and 4.6 ml), and it can be as high as 9 ml after delivery. This finding suggests that the 0.2–12.9 ml of fetal red blood cells in recovered blood was essentially the same amount of fetal red blood cells in maternal circulation after delivery [7, 8]. Moreover, when volume of fetal red blood cells is no more than 4 ml, 500 units of anti-D immune globulin can prevent alloimmunization but within 72 h after delivery [23]. Thus, rapid measurement of the amount of fetal red blood cells in maternal circulation is important [14]. Considering the risk of alloimmunization, patients infused with anti-D immunoglobulin should be followed-up to assess the antibody response in three to six months, while the establishment of a central database to collect summary information is recommended [24].

In this study, the HbF of samples III in the two groups were significantly higher than that in samples I. However, the samples III were nearly the same as the samples IV in the two groups without significant differences compared with the samples I, potentially due to the use of the nonparametric rank correlation test. However, HbF levels of sample III and sample IV were higher in Group 1 than in Group 2, means more anti-D immunoglobulin needed, so two suction devices are recommended to reduce HbF pollution.

### Limitations

Although the sample size was calculated after the pre-test, it only considered the measurement of SCs and AFP. Due to the very low incidence of AFE, a larger sample size is needed to determine the safe use of IOCS in obstetrics.

Thirty-four patients who underwent cesarean section were randomly selected to participate in this study, and the average blood loss was 547 ml. Furthermore, 71% of patients lost less than 350 ml. Due to the small amount of blood loss in some patients, some bias toward lower Hb levels was introduced due to the dilution effect introduced by the recycling machine during the filtration process.

## Conclusions

IOCS combined with a leukocyte filter can significantly reduce the composition of amniotic fluid contained in blood lost during cesarean section,especially in SCs. The amniotic fluid recovered has nearly no effect on the blood filtration effect, but for maternal with Rh-negative blood, we recommend two suction devices to reduce HbF pollution. IOCS in obstetrics still requires further verification in larger samples.

## Data Availability

The datasets used and/or analysed during the current study are available from the corresponding author on reasonable request. (Hong Zeng, yippeerong@139.com).
